# Pressure Support Ventilation During Extracorporeal Membrane Oxygenation Support in Patients With Acute Respiratory Distress Syndrome

**DOI:** 10.1097/MAT.0000000000002285

**Published:** 2024-08-08

**Authors:** Benedetta Fumagalli, Marco Giani, Michela Bombino, Denise Fumagalli, Milena Merelli, Gaia Chiesa, Roberto Rona, Giacomo Bellani, Emanuele Rezoagli, Giuseppe Foti

**Affiliations:** From * Department of Medicine and Surgery, University of Milano-Bicocca, Monza, Italy; † Department of Emergency and Intensive Care, Fondazione Istituto di Ricovero e Cura a Carattere Scientifico San Gerardo dei Tintori, Monza, Italy; ‡ Department of Medical Sciences, University of Trento, Trento, Italy; § Department of Anesthesia and Intensive Care, Santa Chiara Hospital, Azienda Provinciale per i Servizi Sanitari di Trento, Trento, Italy.

**Keywords:** mechanical ventilation, pressure support ventilation, acute respiratory distress syndrome, extracorporeal membrane oxygenation

## Abstract

In the initial phases of veno-venous extracorporeal membrane oxygenation (VV ECMO) support for severe acute respiratory distress syndrome (ARDS), ultraprotective controlled mechanical ventilation (CMV) is typically employed to limit the progression of lung injury. As patients recover, transitioning to assisted mechanical ventilation can be considered to reduce the need for prolonged sedation and paralysis. This study aimed to evaluate the feasibility of transitioning to pressure support ventilation (PSV) during VV ECMO and to explore variations in respiratory mechanics and oxygenation parameters following the transition to PSV. This retrospective monocentric study included 191 adult ARDS patients treated with VV ECMO between 2009 and 2022. Within this population, 131 (69%) patients were successfully switched to PSV during ECMO. Pressure support ventilation was associated with an increase in respiratory system compliance (*p* = 0.02) and a reduction in pulmonary shunt fraction (*p* < 0.001). Additionally, improvements in the cardiovascular Sequential Organ Failure Assessment score and a reduction in pulmonary arterial pressures (*p* < 0.05) were recorded. Ninety-four percent of patients who successfully transitioned to PSV were weaned from ECMO, and 118 (90%) were discharged alive from the intensive care unit (ICU). Of those who did not reach PSV, 74% died on ECMO, whereas the remaining patients were successfully weaned from extracorporeal support. In conclusion, PSV is feasible during VV ECMO and potentially correlates with improvements in respiratory function and hemodynamics.

## Background

One of the major challenges during the acute phase of severe acute respiratory distress syndrome (ARDS) is maintaining adequate oxygenation and carbon dioxide removal while preventing ventilation-induced lung injury.^1^ For this reason, current clinical guidelines suggest the use of neuromuscular blockade and protective mechanical ventilation in the early stages of the disease.^2–5^ As clinical conditions improve, clinicians should contemplate the possibility of transitioning to assisted ventilation.^4,6^ This approach aims to reduce the duration of sedation and muscular paralysis. Prolonged sedation may predispose patients to increased morbidity and adverse events.^7^ Additionally, neuromuscular blockade induces diaphragm dysfunction,^8,9^ whereas early resumption of patient’s spontaneous activity may prevent muscle atrophy and contribute to the healing process.^10^

In the most severe cases, when the derangement in gas exchanges can no longer be corrected through lung-protective invasive ventilation, rescue therapies such as veno-venous extracorporeal membrane oxygenation (VV ECMO) may be required.^3^ With the assistance of VV ECMO, the protective ventilatory strategy can be further optimized into an “ultra-protective” approach.^11^ While on ECMO, if gas exchange and respiratory mechanics improve, assisted mechanical ventilation can be considered, for the reasons mentioned earlier. Moreover, the essential role of the extracorporeal support in controlling respiratory drive through carbon dioxide removal may facilitate and accelerate the transition to assisted ventilation.^12,13^

The primary objective of this investigation was to assess the feasibility of transitioning to pressure support ventilation (PSV) during VV ECMO. As a secondary objective, we aimed to report the variations in respiratory mechanics, oxygenation parameters, and hemodynamics following the transition to PSV.

## Materials and Methods

We designed a retrospective monocentric study, including all consecutive adult patients with severe ARDS treated with VV ECMO in the intensive care unit (ICU) of IRCCS San Gerardo dei Tintori between September 2009 and October 2022.

In accordance with local ethical standards, no informed consent was required for this retrospective, observational study. The Comitato Etico Brianza (ref. NP3369) approved the data collection for this study.

### Patient Management

At our institution, severe ARDS patients requiring VV ECMO are managed in the early phase after cannulation on controlled mechanical ventilation (CMV) (pressure-controlled ventilation or volume-controlled ventilation) with deep sedation, neuromuscular blockers, and prone positioning.^14^ Volatile sedation is often used during this phase.^15^ After a few days, if and when clinical conditions improve, and the respiratory mechanics become compatible with spontaneous breathing, neuromuscular blockers are discontinued, and sedation level is adjusted to facilitate the transition to assisted ventilation. Typically, PSV mode is employed: 100% of tidal breaths spontaneously triggered by the patient, with the frequent use of a sigh breath (set at 30–35 cm H_2_O for 3–3.5 seconds) every 2 minutes. The ECMO sweep gas flow rate is adjusted to control the respiratory drive and to facilitate spontaneous breathing. Extracorporeal membrane oxygenation weaning is then initiated with the patient on PSV.

### Data Collection

We conducted a retrospective data collection from patients’ electronic records obtaining epidemiological and baseline characteristics, including age, gender, body mass index (BMI), main comorbidities, causes of ARDS, and ratio of arterial oxygen tension to inspiratory oxygen fraction (PaO_2_/FiO_2_) and Sequential Organ Failure Assessment (SOFA) score before ECMO start. We considered only the first attempt of PSV lasting at least 24 hours during the first ECMO run for each patient. We deemed the PSV trial successful if assisted ventilation was sustained for a minimum of 72 consecutive hours on ECMO or if the patient was weaned from ECMO within 72 hours while still on PSV. “We collected data at two-time points: in the morning on the final day of CMV and approximately 24 hours after the initiation of PSV, with a variability of ±6 hours, ranging between 18 and 30 hours.” The data gathered at these time points were the following:

- Respiratory parameters, namely mechanical ventilation settings (tidal volume, respiratory rate, minute ventilation, positive end-expiratory pressure [PEEP], pressure support level), respiratory mechanics (respiratory system compliance, driving pressure, Pressure Muscle Index [PMI], occlusion pressure at 100 msec—P_0.1_), and gas exchange (pulmonary shunt fraction, PaO_2_, FiO_2_, arterial carbon dioxide tension—PaCO_2_, and pH).
- Hemodynamic parameters: cardiac output, wedge pressure, preoxygenator saturation of oxygen (SpreO_2_) (a surrogate for central venous saturation in ECMO patients), heart rate, systemic and pulmonary arterial pressures, and cardiovascular (CV) SOFA score.^16^
- Intravenous sedatives with their dosage.
- Extracorporeal membrane oxygenation settings, including blood flow, sweep gas flow rate and oxygen fraction, and rotations per minute of the blood pump.

Pulmonary shunt fraction, indicated as the ratio of the blood flow that is not subject to gas exchange (*Qs*) to the total cardiac output (*Qt*), was calculated using the following formula^17^


$$\begin{array}{l} \frac{Qs}{Qt}=\frac{Ccap-Cart}{Ccap-Cven} \end{array}$$

where Ccap represents the oxygen content of blood from lung capillaries after gas exchange, Cart represents the oxygen content in the arterial blood and Cven is the oxygen content in the venous blood from the pulmonary arteries.

According to clinical practice at our facility, pulmonary shunt fraction is measured with 100% FiO_2_.^18^

We employed pulmonary artery catheters that allow continuous cardiac output monitoring, to overcome the inaccuracy of the intermittent bolus thermodilution technique described in patients on extracorporeal support.^19^ With the continuous thermodilution technique, in fact, the change in blood temperature occurs in the right ventricle^20,21^ and not in the central venous circulation (were cold/room temperature boluses are typically injected with the intermittent thermodilution method), and so the “indicator” is far less likely to be drawn into the extracorporeal circuit.

### Statistical Analysis

Numerical data were presented as median (interquartile range [IQR]) and categorical data as count (percentage). In patients who underwent a PSV trial, we compared parameters at two specified timepoints (*ie*, before and after the transition to assisted PSV) by means of a matched pair analysis (Wilcoxon signed-rank test). Patients were stratified based on the success or failure of the PSV trial. Characteristics of the two subgroups, defined by the PSV trial outcome, were compared using the Wilcoxon test for continuous variables, and the Fisher’s exact test or Pearson Chi-Square test for categorical variables, as deemed appropriate.

A two-tailed *p* value of less than 0.05 was considered statistically significant. Statistical analysis was performed using JMP 16.0 software (SAS, Cary, NC).

## Results

**Figure 1.**
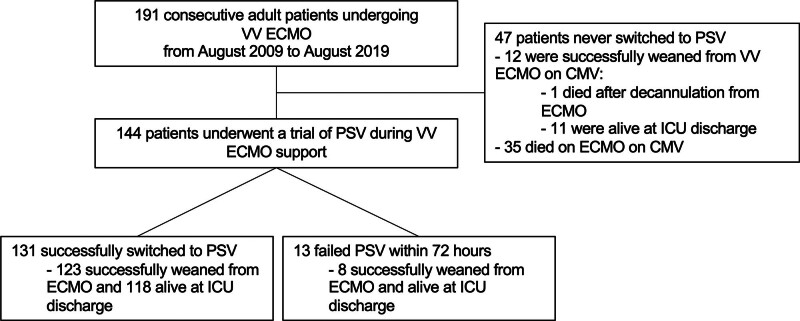
Flowchart illustrating the study population. CMV, controlled mechanical ventilation; ICU, intensive care unit; PSV, pressure support ventilation; VV ECMO, veno-venous extracorporeal membrane oxygenation.

Over the study period, 191 patients met the inclusion criteria and were included in the analysis. Study flowchart is presented in Figure 1. Seventy-two (38%) were females, median age was 51 (43–59) years old, BMI was 28 (25–33) kg/m^2^. The leading cause of ARDS was viral pneumonia (n = 97, 51%), of which 46 (47%) were caused by SARS-CoV-2, followed by bacterial pneumonia (n = 62, 32%), autoimmune disease (n = 12, 6%), and trauma (n = 6, 3%). Main comorbidities were hypertension (n = 58, 30%), immunodeficiency (n = 45, 24%), and diabetes (n = 29, 15%). Overall mortality assessed at ICU discharge was 28%.

Forty-seven (25%) patients did not reach the PSV trial breathing due to excessive severity of illness. This subgroup of patients showed a very high ICU mortality (77%).

One hundred and forty-four (75%) patients had a trial of PSV during ECMO support. The median duration of CMV on ECMO before the pressure support trial was 9 (5–15) days. Sigh breath was used in 91 (69%) of these patients during PSV.

Table 1 shows mechanical ventilation and ECMO settings, respiratory mechanics, gas exchange, and hemodynamics during the last day of CMV and the first day of PSV.

**Table 1.** Respiratory Parameters, ECMO Settings, and Hemodynamics and Before and After Transitioning From CMV to PSV

N = 144	CMV	PSV	*p* Value
Respiratory parameters			
Tidal volume (ml)	300 (260–380)	350 (290–450)	<0.001
Respiratory rate	10 (10–12)	15 (12–18)	<0.001
Minute ventilation (L/min)	3.2 (2.7–4.0)	5.1 (4.1–6.7)	<0.001
Blood flow (L/min)	3.4 (3.1–3.7)	3.2 (3.0–3.6)	**0.008**
Sweep gas flow (L/min)	5.0 (4.0–6.0)	4.5 (3.0–6.0)	<0.001
FiO_2_ (%)	50 (45–60)	50 (40–60)	0.002
PEEP (cm H_2_O)	15 (13–16)	14.5 (12–16)	<0.001
Pressure support (cm H_2_O)	—	10 (10–12)	—
PMI (cm H_2_O)	—	0 (0–2)	—
P_0.1_ (cm H_2_O)	—	−0.7 (−1.5 to 0)	—
Driving pressure (cm H_2_O)	9 (8–10)	10 (9–12)	<0.001
Respiratory system compliance (ml/cm H_2_O)	33 (26–44)	38 (27–47)	0.002
PaO_2_ (mm Hg)	84 (76–93)	85 (77–97)	0.645
PaCO_2_ (mm Hg)	49 (44–54)	45 (42–49)	<0.001
pH (mm Hg)	7.42 (7.39–7.44)	7.44 (7.41–7.46)	<0.001
Pulmonary shunt fraction (%)	40 (32–49)	38 (28–46)	<0.001
Hemodynamics			
SpreO_2_ (%)	83 (78–87)	82 (78–87)	0.451
Cardiac output (L/min)	7.6 (6.5–9.0)	7.5 (6.5–9.1)	0.740
Wedge pressure (mm Hg)	12 (10–14)	12 (10–15)	0.676
Central venous pressure (mm Hg)	10 (8–13)	10 (7–12)	0.009
Heart rate (bpm)	94 (83–105)	92 (80–108)	0.327
Systolic arterial pressure (mm Hg)	123 (112–135)	134 (118–149)	<0.001
Mean arterial pressure (mm Hg)	81 (72–89)	83 (74–95)	0.005
Diastolic arterial pressure (mm Hg)	60 (52–65)	62 (56–70)	0.012
Systolic pulmonary pressure (mm Hg)	35 (30–42)	33 (28–41)	0.044
Mean pulmonary pressure (mm Hg)	26 (22–30)	25 (20–29)	0.023
Diastolic pulmonary pressure (mm Hg)	18 (15–22)	17 (13–21)	0.020

Data are expressed as median (interquartile range).

BPM, beats per minute; ECMO, extracorporeal membrane oxygenation; FiO_2_, inspired fraction of oxygen; P_0.1_, occlusion pressure at 100 msec; PaCO_2_, arterial tension of carbon dioxide; PaO_2_, arterial tension of oxygen; PEEP, positive end expiratory pressure; PMI, pressure muscle index; SpreO_2_, preoxygenator hemoglobin saturation of oxygen.

### Respiratory Parameters

While transitioning to assisted ventilation, sweep gas flow rate was slightly decreased and minute ventilation at the natural lung almost doubled. Relatively high PSV levels were used, with minimal patient effort, as shown by PMI and P_0.1_ values. Median tidal volume and respiratory rate were higher on PSV, as compared to CMV. A significant reduction of PaCO_2_ and pH was concurrently recorded. Shunt of the natural lung was significantly decreased, and respiratory system compliance significantly improved after 24 hours of PSV.

### Hemodynamic Parameters

**Figure 2.**
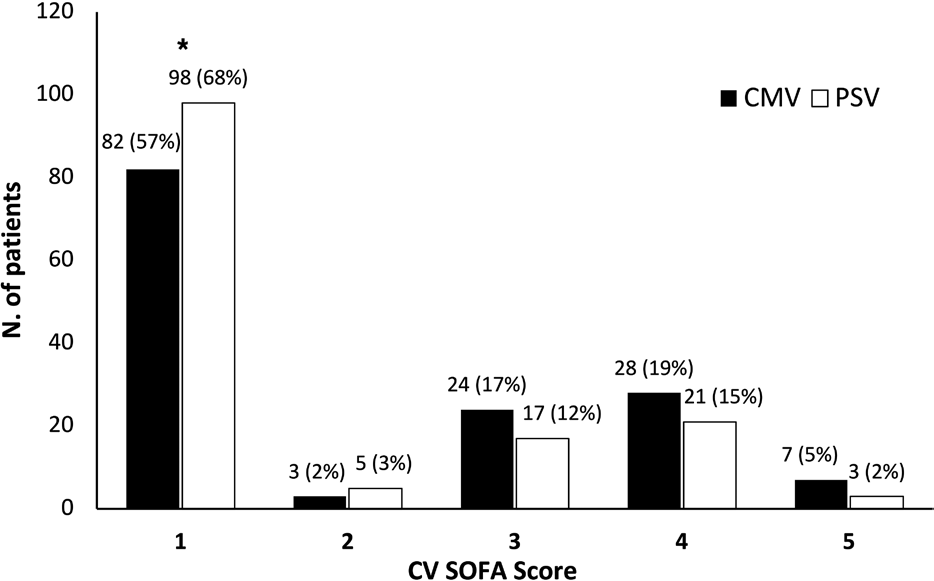
CV SOFA score during CMV and PSV. CV SOFA score: 0 = MAP >70 mm Hg without catecholamines; 1 = MAP <70 mm Hg without catecholamines; 2 = dopamine ≤ µg/kg min^−1^ or dobutamine any dose; 3 = dopamine >5 mcg/kg/min or epinephrine ≤0.1 µg/kg min^−1^ or norepinephrine ≤0.1 µg/kg min^−1^; 4 = dopamine >15 µg/kg min^−1^ or epinephrine >0.1 µg/kg min^−1^ or norepinephrine >0.1 µg/kg min^−1^. % refers to the total number of patients that experienced a trial of PSV (144). *p < 0.05 *vs*. CMV. CMV, controlled mechanical ventilation; CV SOFA, Cardiovascular Sequential Organ Failure Assessment; MAP, mean arterial pressure; PSV, pressure support ventilation.

After transitioning to PSV, systemic arterial pressures were significantly higher than on CMV. Accordingly, the proportion of patients with a mean arterial pressure above 70 mm Hg without any vasopressor or inotrope (*ie*, CV SOFA score = 0, see Figure 2) was significantly higher (68% *vs*. 57%, respectively, *p* = 0.040). Pulmonary arterial pressures were slightly lower on the first day of PSV, whereas cardiac output did not vary.

### Sedation Management

Details on sedation management before and after the transition to PSV are provided in Table S1, Supplemental Digital Content, http://links.lww.com/ASAIO/B315. A decrease in the infusion rates of all intravenous sedative drugs was recorded. Neuromuscular blocking agents were administered in 121 patients (84%) during CMV. Two (2%) patients received a low dose of neuromuscular blockers during the first day of assisted ventilation.

### Successful *Versus* Failed Transition to Pressure Support Ventilation

Thirteen patients (9%) required to be switched back to CMV within 72 hours from the initiation of PSV, whereas 133 patients (91%) remained on PSV. A comparison of patients who underwent a successful versus unsuccessful PSV trial is presented in Table S2, Supplemental Digital Content, http://links.lww.com/ASAIO/B315. Baseline characteristics did not differ between the two subgroups. A high rate of successful ECMO weaning and a low ICU mortality were recorded in the successful PSV group, together with a significantly shorter ECMO duration.

Table S3, Supplemental Digital Content, http://links.lww.com/ASAIO/B315, presents a comparison of hemodynamic and respiratory parameters before and after the transition to PSV, after stratification based on success or failure of the PSV trial. Respiratory variables demonstrated significant improvement exclusively among patients who successfully completed the PSV trial. Conversely, those who failed the PSV trial exhibited a trend toward worsened pulmonary shunt fraction and respiratory system compliance, although statistical significance was not achieved. Of note, pulmonary shunt fraction on PSV was significantly lower in those who succeeded as compared to those who failed the PSV trial.

## Discussion

To our knowledge, this study is the first to examine the feasibility of transitioning from CMV to PSV during VV ECMO, simultaneously evaluating the impact of this shift on respiratory parameters and hemodynamics. In a cohort of patients with severe ARDS necessitating extracorporeal support, a majority successfully transitioned to PSV during ECMO. Those who completed this transition were likely to be weaned from ECMO and discharged from the ICU. Transitioning to assisted mechanical ventilation was associated with improvements in respiratory mechanics, pulmonary shunt, and hemodynamics, as compared to controlled ventilation.

The optimal ventilatory strategy for patients undergoing VV ECMO, a rescue therapy for severe ARDS, has not been defined. As of now, no standard of care exists, as no clinical trial has ever compared different ventilatory strategies during extracorporeal support.^11^ Following the guidance in the dedicated Extracorporeal Life Support Organization (ELSO) guidelines, ventilator settings should align with those reported in the two largest and most recent ECMO trials to date^22^: the EOLIA trial^23^ and the CESAR trial.^24^ Ventilatory practices in these trials aim for a plateau pressure not exceeding 24 cm H_2_O, a PEEP of 10 cm H_2_O or higher, a respiratory rate of 10 breaths per minute (up to 30 if necessary), and an FiO_2_ ranging from 0.3 to 0.5.^23,24^ However, it is straightforward that the ventilator management should be adjusted as the clinical condition changes.

In this study, we observed a significant improvement in respiratory parameters after transitioning from CMV to PSV. First, pulmonary shunt fraction was significantly reduced. This is likely attributable to the improvement of ventilation-perfusion matching and to the recruitment of dependent lung regions, resulting in an increase of functional residual capacity (FRC).^25,26^ Accordingly, whereas acknowledging the limitations previously specified by our group,^27^ respiratory system compliance was increased. The observed rise in driving pressure following the transition to PSV, despite an increase in respiratory system compliance, can be hence attributed to the higher tidal volumes. In this light, it is crucial to emphasize the importance of maintaining a protective ventilation strategy even after the transition to assisted ventilation. Within our cohort, although the driving pressure slightly increased, it remained consistently below the “accepted” thresholds.^28^ Minute ventilation also increased, suggesting a rise in metabolic demand as patients commence efforts to trigger the ventilator and initiate spontaneous breathing. As reported by Cereda *et al.*,^6^ these changes may also be attributed to an attempt to reduce CO_2_ levels in patients who were mildly hypercapnic during controlled ventilation, even with normal pH. In contrast to the findings by Cereda *et al.*,^6^ the transition to assisted spontaneous breathing did not lead to alkaline pH values. Extracorporeal support offers a means to control and modulate the respiratory drive in spontaneous breathing patients by titrating CO_2_ removal.^12,13^ Finally, it’s worth noting that the reduction in sedative dosages, which may uncover an anxious state and heighten pain sensations, could have contributed to increased respiratory drive, resulting in a higher respiratory rate and tidal volume.

After switching to assisted ventilation, an improvement in systemic hemodynamics was recorded. This can be likely due to the interaction of two factors: the reduction of intrathoracic pressure facilitating venous return, and a slightly lower sedation, resulting in an increased systemic vascular tone. Additionally, a slight reduction in pulmonary arterial pressures was noted, potentially attributed to the decrease in arterial carbon dioxide levels. Although the magnitude of this phenomenon was modest, it hints at a potential role of assisted breathing in unloading the right heart.

The findings of the current study suggest that maintaining a “gentle”^26,29^ spontaneous breathing might be a way to improve the respiratory function during ECMO support, and potentially enhance the healing process and facilitating the weaning from the extracorporeal support. Additionally, interventions leading to a reduction of sedatives and the discontinuation of neuromuscular blockade may hold the potential to improve clinical outcomes.^30^ We acknowledge that some changes in respiratory mechanics may be attributed to sedation reduction, which might not be evident under CMV. However, one of the advantages of PSV is indeed the allowance for lighter sedation. This further reinforces our belief that transitioning to pressure spontaneous ventilation could be beneficial. Regarding the specific issue of neuromuscular blockade, we report two patients were transitioned to PSV still under low-dose neuromuscular blocking agents: this may represent an interesting compromise between the unmissable goal of lung-protective ventilation and fast liberation from neuromuscular blockade, as described by Doorduin *et al*.^31^ However, potential harms of assisted ventilation in such delicate patients should be always kept in mind, one above all the risk of patient self-induce lung injury (PSILI).^26,29,32^ For this reason, we surely recommend careful and close monitoring of respiratory mechanics in patients tackling this crucial transition from controlled ventilation to assisted spontaneous breathing. In any case, it should be noted that whether assisted spontaneous breathing predominantly offers benefits or risks may depend on the patient’s respiratory pattern and its interaction with the ventilator, its biological predisposition to mechanical injury, and the phase and duration of ARDS.^29^

The relatively small proportion of patients failing the PSV was likely due to the approach of the attending clinicians in the ICU, waiting for gas exchange and respiratory mechanics to recover before attempting a PSV trial. This hindered the feasibility of conducting specific analyses aimed at defining predictors of PSV failure.^33,34^ However, no significant baseline differences that could predict PSV failures were observed between the patients who tolerated PSV and those who failed. Nonetheless, drawing conclusions on this matter is challenging due to the significant disparity in group sizes, with 131 patients having successful PSV trials compared to only 13 patients with unsuccessful trials.

This study presents some limitations. First, as clinical improvement is a fundamental prerequisite for initiating ventilatory weaning,^4,6^ the patients who were not successfully transitioned to PSV were inherently sicker than those in the PSV group. Therefore, selection bias was unavoidably present when comparing the two groups. From this perspective, we cannot exclude the possibility that improvements in respiratory mechanics and hemodynamics were secondary to the natural healing process of the patients. Second, the study relied on retrospective data collection, and the decisions to transition to PSV and the return to CMV were based on clinical evaluations by physicians rather than standardized, preestablished criteria. Additionally, as a monocentric study, the generalizability of the results to broader populations may be limited. Third, we focused solely on the first day of assisted ventilation to observe the physiological effects during this early phase of spontaneous breathing resumption. Fourth, inspiratory effort was evaluated noninvasively at bedside using P_0.1_ and PMI; however, data on expiratory occlusion pressure were not available.^35^ Lastly, although we recorded parenteral sedation levels, a comprehensive understanding of the sedative plan was impeded by missing data on enteral sedation and by the absence of a systematic assessment employing a validated and universally recognized scale (such as the Richmond Agitation Sedation Scale [RASS], which is the most frequently used scale at our institution).

## Conclusions

In ARDS patients on extracorporeal respiratory support, the transition to PSV was achieved in the majority of patients, and was associated to improvements in respiratory function and hemodynamics. Further prospective investigations, involving a larger patient sample and a standardized protocol guiding clinicians in the decision to switch to assisted mechanical ventilation, are warranted to better characterize the timing and identify patients who can benefit from spontaneous breathing during VV ECMO.

## Supplementary Material


