# Supplementary material for: Pressure Support Ventilation During Extracorporeal Membrane Oxygenation Support in Patients With Acute Respiratory Distress Syndrome
Source: ASAIO J. 2024 Aug 8;71(2):171–6. doi: 10.1097/MAT.0000000000002285 (PMC11761049; doi:10.1097/MAT.0000000000002285)
Supplement: Supplementary file 1 [file mat-71-171-s001.pdf]

Supplemental Digital Content

Pressure Support Ventilation during Extracorporeal Membrane Oxygenation support in patients with Acute Respiratory Distress Syndrome

Benedetta Fumagalli, Marco Giani, Michela Bombino, Milena Merelli, Gaia Chiesa, Roberto Rona, Giacomo Bellani, Emanuele Rezoagli and Giuseppe Foti

SUPPLEMENTARY RESULTS

	CMV	PSV	P value
Propofol, mg/h	220 [160-300]	200 [160-300]	**0.023**
N	83	123	
Isoflurane, ET%	1.0 [0.9-1.1]	-	-
N	43		
Fentanyl, mcg/h	100 [100-100]	100 [54-100]	**<0.001**
N	131	120	
Remifentanil, mcg/kg/min	0.21 [0.08-0.35]	0.11 [0.07-0.15]	1
N	4	18	
Midazolam, mg/h	4 [3-7]	4 [3-5]	**<0.001**
N	41	42	

Table S1. Comparison of sedative setting controlled mechanical ventilation (CMV) and pressure support ventilation (PSV). ET %, end tidal concentration.

	PSV trial Successful (n= 131)	PSV trial Unsuccessful (n=13)	P value
Age, yr	51 (43-58)	51 (39-60)	0.967
Females, n	51 (39%)	5 (38%)	0.974
BMI, kg/m^2	29 (25-33)	27 (24-31)	0.228
SOFA score before ECMO	7 (5-10)	8 (6-14)	0.253
PaO_2/FiO_2 before ECMO, mmHg	74 (60-90)	77 (50-108)	0.585
Respiratory system compliance before ECMO, ml/cmH_2O	30 (23-37)	29 (21-38)	0.903
MV duration before ECMO start, days	2 (1-5)	3 (2-7)	0.090
Causes of ARDS			
Pneumonia	115 (88%)	12 (92%)	0.630
Viral	63 (48%)	6 (46%)	
Bacterial	42 (32%)	6 (46%)	
Unknown	10 (8%)	0	
Trauma	5 (4%)	0	0.473
Autoimmune disease	9 (7%)	1 (8%)	0.911
Septic shock	2 (2%)	0	0.654
**OUTCOMES**			
Successful ECMO weaning	123 (94%)	8 (62%)*	<0.001
ECMO duration, days	14 (9-21)	19 (13-38)*	0.036
ICU length of stay, days	29 (21-50)	45 (30-54)	0.121
ICU outcome, death	13 (10%)	5 (38%)*	0.003

Table S2. Characteristics and outcomes of the study population stratified based on the outcome of PSV trial. Data are expressed as absolute number (%) or median (Interquartile range). BMI, body mass index; SOFA, sequential organ failure assessment; MV, mechanical ventilation; COPD, chronic obstructive pulmonary disease; ICU, intensive care unit.
*=p<0.05.

	Successful PSV trial (n= 131)		Unsuccessful PSV trial (n= 13)	
	CMV	PSV	CMV	PSV
Driving pressure, cmH$_2$O	9 [8-10]	10 [9-12]*	9 [8-11]	10 [8-14]
PEEP, cmH$_2$O	15 [13-16]	14 [12-16]*	15 [13-15]	15 [13-16]
Pressure Support, cmH$_2$O	-	10 [10-12]	-	10 [10-11]
PMI, cmH$_2$O	-	0 [0-2]	-	0 [0-0]
$P_{0.1}$, cmH$_2$O	-	–0.7 [–1.5-0]	-	–0.5 [–2- 0.3]
Respiratory system compliance, ml/cmH$_2$O	33 [26-44]	38 [27-47]*	34 [22-44]	34 [23-46]
Tidal volume, ml	300 [260-380]	360 [300-450]*	300 [230-380]	340 [205-400]
Respiratory rate	10 [10-12]	15 [12-18]*	10 [10-13]	16 [13-23]*
Minute ventilation, l/min	3.2 [2.7-4.0]	5.2 [4.2-6.8]*	3.2 [2.6-4.0]	4.3 [3.5-7.3]*
FiO_2, %	50 [40-60]	50 [40-55]*	50 [50-80]	55 [45-65]
P_aO_2, mmHg	85 [77-93]	85 [78-97]	75 [71-92]	85 [68-98]
p_aCO_2, mmHg	49 [44-53]	45 [41-49]*	50 [46-56]	45 [42-51]
pH	7.42 [7.40-7.44]	7.44 [7.41-7.46]*	7.42 [7.39-7.43]	7.45 [7.40-7.47]
Pulmonary Shunt fraction, %	40 [32-49]	35 [28-45]*§	44 [32-57]	49 [37-60]
SpreO2, %	83 [78-87]	82 [78-87]	81 [78-88]	77 [74-87]
Cardiac output, l/min	7.4 [6.4-9.1]	7.6 [6.5-9.1]	7.4 [6.7-8.4]	7.3 [6.9-8.9]
Wedge pressure, mmHg	12 [10-15]	12 [9-15]	12 [10-13]	12 [10-12]
Heart rate, bpm	93 [83-105]	91 [80-107]	100 [85-113]	91 [80-107]
Systolic Arterial Pressure, mmHg	123 [111-135]	134 [117-150]*	129 [115-145]	138 [118-146]
Mean Arterial Pressure, mmHg	80 [71-87]	83 [74-95]*	82 [74-96]	83 [78-96]
Diastolic Arterial Pressure, mmHg	60 [51-65]	62 [56-71]*	62 [55-71]	67 [57-71]
Systolic pulmonary pressure, mmHg	36 [30-42]	34 [29-41]	32 [29-42]	32 [28-38]
Mean pulmonary pressure, mmHg	18 [15-23]	18 [13-21]*	17 [13-20]	16 [14-20]
Diastolic pulmonary pressure, mmHg	26 [22-30]	25 [21-29]*	23 [21-31]	23 [19-26]

Table S3. Respiratory parameters and hemodynamics before and after transitioning from controlled mechanical ventilation (CMV) to pressure support ventilation (PSV). FiO2, inspired fraction of oxygen; PEEP, positive end expiratory pressure; PMI, pressure muscle index; P0.1, occlusion pressure at 100 msec; PaO2, arterial tension of oxygen; PaCO2, arterial tension of carbon dioxide; SpreO2, pre oxygenator saturation of oxygen; BPM, beats per minute *$p<0.05$ vs CMV, §$p<0.05$ vs failure, same timepoint.
